# Properties of Red Mud Neutralized with Sulfuric Acid and Effects on Cement Mortar

**DOI:** 10.3390/ma16134730

**Published:** 2023-06-30

**Authors:** Suk-Pyo Kang, Sang-Jin Kim, Seong-Uk Hong, Byoung-Ky Lee

**Affiliations:** 1 Department of Construction Engineering, Woosuk University, Jincheon 27841, Republic of Korea; ksp0404@woosuk.ac.kr (S.-P.K.);; 2 Department of Architecture, Woosuk University, Jincheon 27841, Republic of Korea; 3 COCHEMS Co., Ltd., Industrial Tools Circulating Center, 160, Daehwa-ro, Daedeok-gu, Daejeon 34368, Republic of Korea

**Keywords:** red mud, liquefied red mud, neutralized red mud, cement mortars, recycling, X-ray diffraction, SEM

## Abstract

The purpose of this study was to recycle red mud, an industrial byproduct that generates 300,000 tons per year, into the construction industry. Red mud was prepared as a liquid, neutralized with sulfuric acid, and replaced with cement mortar. The properties of liquefied red mud (LRM) neutralized with sulfuric acid (LRM + S) were investigated as well as its effect on cement mortar’s mechanical and hydration characteristics. The pH of LRM + S stabilized at 7.6; its SO_3_ content was ~4.19% higher than that of LRM. Sulfites were contributed by calcium and sodium sulfate. The flows and setting times of the mortars containing LRM and LRM + S decreased as the substitution rate increased. The compressive strength of mortar that replaced 5% of cement with LRM + S was similar to that of the plain cement mortar. Scanning electron microscopy and X-ray diffraction revealed that the hydration products of LRM + S-containing cement mortar were similar to those of the plain cement mortar. Thus, LRM + S can be used as a cement substitute.

## 1. Introduction

Portland cement is currently considered to be the dominant building material [1]. In addition, concrete, made from Portland cement, water, and aggregates, is the second most used substance after water in the world [2]. For CO_2_ released from fossil consumption and cement production are the main anthropogenic contributor to global warming. Portland cement production causes high CO_2_ emis- 35 sions, accounting for 7–10% of global anthropogenic CO_2_ emissions and 2–3% of energy consumption [3,4]. Therefore, in order to reduce CO_2_ emissions from the cement industry, red mud, an industrial waste, was proposed to be used as a substitute for cement.

Red mud is a reddish-brown waste product generated by the aluminum extraction process from bauxite ore. It mainly consists of iron oxide and is highly alkaline [5,6]. In 2021, approximately 170 million tons of red mud waste was generated owing to increased aluminum production. The amount of red mud waste is expected to increase continuously with the increasing aluminum consumption [7,8]. Red mud is difficult to use because of its highly alkaline nature; therefore, most of it is stored under wet or dry conditions or discarded. Wet storage causes sewage-associated contamination of surface water and soil salinization because the alkali in red mud slurry penetrates the ground [9]. Dry storage causes significant atmospheric pollution because red mud generates particulate matter [10]. In order to solve the environmental problems and limited space related to the storage and treatment of red mud, many studies have been conducted to apply industrial waste red mud to the construction industry by using cement-based materials that are generally inexpensive and easily available [11].

For example, a study proposed using red mud in producing construction materials as an eco-friendly and efficient approach [12]. Ghalehnovi et al. evaluated the properties of self-compacting concrete that contained red mud as a substitute for cement and a filler [13]. They reported that this substitution had a negligible adverse effect on the performance of concrete and could improve the sulfate resistance of concrete. Senff et al. [14] investigated the rheological and strength characteristics of concrete containing red mud and reported that red mud does not affect the concrete hydration process. However, they also reported that the red mud content in concrete exceeds 20 wt.%, absorbed water increases, and compressive strength decreases. Liu et al. [15] reported that adding red mud to concrete can improve its compressive strength. Further, adding red mud to magnesium phosphate cement increased its strength and water resistance and decreased porosity. Some components of red mud can form new hydrates with cementitious properties through reactions with magnesium phosphate cement. However, as red mud has a high Na_2_O content and is thus highly alkaline, using it in building materials on a large scale is not easy [16].

Most studies on the recycling of red mud have used powdered red mud produced through a dry grinding process. However, this recycling process is complex and economically inefficient. The red mud’s recycling rate has typically stayed within 10% [17]. In previous studies, liquefied red mud (LRM) was neutralized with sulfuric acid (LRM + S) or nitric acid and was added to cement paste to improve the economic efficiency and recycling rate [18,19]. The initial compressive strength of cement paste containing LRM + S was >85% of that of the plain cement paste, and their compressive strengths at 28 days of aging were similar. The possibility of commercializing LRM + S was also proposed because the hydration heat and hydration products were similar to those of the plain cement paste. For the commercialization of LRM + S as a construction material, further research on its effects on mortar and concrete is required [20]. 

In this study, in an attempt to improve the strength degradation observed earlier, we neutralized LRM to reduce its high pH (from 10–12 to 7–8). For experimental validation, we compared added LRM and neutralized LRM (LRM + S) to cement mortar and examined the heat of compressive strength and hydration products of the samples using X-ray diffraction (XRD) and SEM. We expect the results of our study to contribute to an increase in the recycling of red mud as a construction material.

## 2. Materials and Methods

### 2.1. Materials

Table 1 shows the physical and chemical properties of red mud sludge (KC Co., Ltd., Seoul, Republic of Korea) used to produce LRM and LRM + S. The main chemical components of red mud sludge are SiO_2_, Al_2_O_3_, and Fe_2_O_3_; they account for approximately 80 wt.%. Reagent-grade sulfuric acid (95% purity, Daejung Chemicals & Metals Co., Ltd., Siheung-si, Republic of Korea) neutralized the LRM. The cement used (Sungshin Cement Co., Ltd., Seoul, Republic of Korea) was Ordinary Portland Cement (OPC); its physical and chemical properties are shown in Table 2. The fine aggregate had natural spherical particles with a maximum diameter of 1.6 mm and a minimum silicon dioxide content of 98%.

Table 3 lists the properties of the dispersant (polycarboxylic-acid-based, company S) and thickener (methyl-cellulose-based, company H) used to produce the LRM. The anti-foaming agent (polyoxyalkylene-alkylether-based, company K) was a transparent liquid with a pH value of 4.5–7.5, a specific gravity of 0.9984, and a viscosity of 269 cP (25 °C).

### 2.2. Methods

#### 2.2.1. Sample Preparation 

**Figure 1:**
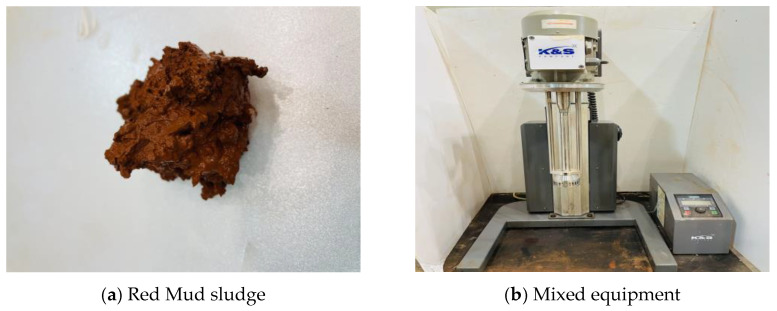

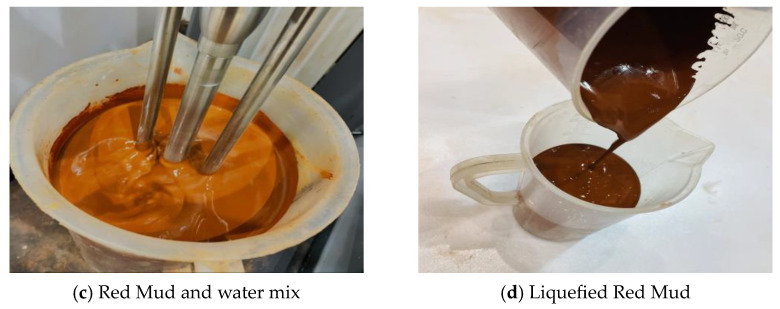
Manufacturing process of Liquefied Red Mud.

LRM was produced by mixing red mud sludge with water, the dispersant, and the antifoaming agent in a mass ratio of 1:0.2:0.0036:0.0014. Red mud sludge and water were first stirred at 20,000 rpm for 3 min using a Homo Mixer (K & S Co., Mokpo, Republic of Korea), as shown in Figure 1, and the dispersant and antifoaming agent were then added at the above ratio and stirred for 2 min [21]. The physical and chemical properties of the resulting LRM were analyzed. LRM + S was produced by mixing 3.4 or 6.8 g of sulfuric acid with 100 g of LRM. The pH change of the mixture over time was measured using a pH meter (HI-991300, Hanna Instruments, Woonsocket, RI, USA) between 10 min and 12 h after adding the sulfuric acid. The amount of sulfuric acid needed to maintain a pH value of 7–8 for up to 12 h was determined. The mineral composition of the LRM + S was analyzed using X-ray diffraction. Samples were then dried in an oven at 105 °C for 24 h. The dried samples were pulverized and sifted through a 200-mesh sieve.

Table 4 shows the mix design of cement mortar containing LRM and LRM + S as cement substitutes. For the mixes of cement mortar replacing 5 and 10 wt.% of cement with LRM and LRM + S, the water content was adjusted to ensure uniform mixing. Cement mortars were mixed for 4 min using a mortar mixer (Mortar Mixer, Heungjin, Republic of Korea). The cement mortars containing 5 and 10 wt.% LRM are henceforth termed LM5 and LM10, respectively. The cement mortars containing 5 and 10 wt.% LRM + S are henceforth termed SRM5 and SRM10, respectively.

#### 2.2.2. Testing Methods

The flow was measured according to the ASTM C1437 [22] test method (Standard Test Method for the Flow of Hydraulic Cement Mortar). The base diameter of the mortar was measured four times at equal intervals, and the average value was used for the flow calculation.

The setting time was measured by the ASTM C191 [23] test method (Standard Test Method for Time of Setting of Hydraulic Cement by Vicat Needle).

The compressive strength was measured as per the ASTM C349 [24] test method (Standard Test Method for Compressive Strength of Hydraulic Cement Mortars) using a universal testing machine (Heungjin Testing Machine Co., Ltd., Gimpo-si, Republic of Korea). The specimens each had a size of 40 × 40 × 160 mm^3^ and were cured at a temperature of 20 ± 2 °C and a relative humidity of 50%. The compressive strengths were measured at 1, 3, 7, and 28 days of aging. The reported compressive strength results are average values taken from three mixed samples. 

For microstructural analysis, cement mortar samples at 1 and 28 days of aging were collected and immersed in anhydrous ethanol for 24 h to stop hydration. They were then dried in an oven at 40 °C for 24 h. The dried samples were observed using cold-type field emission SEM (S-4800, Hitachi, Tokyo, Japan).

To analyze hydration products, samples at 1 and 28 days of aging were collected and immersed in anhydrous ethanol for 24 h to stop hydration. They were then dried in an oven at 40 °C for 24 h. The dried samples were pulverized, sifted through a 200-mesh sieve, and analyzed via XRD (SmartLab, Rigaku, Tokyo, Japan) [25]. XRD analysis was performed using a CuKa wavelength at 45 kV and 200 mA and at 4°/min in the range of 2θ = 5–75°.

## 3. Results and Discussion

### 3.1. Properties of LRM + S

3.1.1. pH

**Figure 2:**
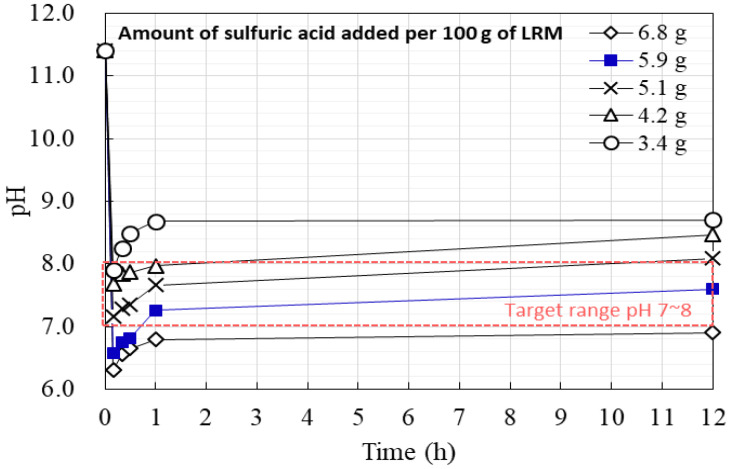
pH changes of LRM + S.

To determine the appropriate amount of sulfuric acid needed to produce LRM + S, varying amounts of sulfuric acid were added per 100 g of LRM, and pH values were measured over 12 h. As shown in Figure 2, initial pH values decreased with increasing amounts of sulfuric acid but tended to increase over time. The optimal amount of sulfuric acid needed to maintain the pH between 7 and 8 for 12 h was found to be 5.9 g.

#### 3.1.2. XRD

**Figure 3:**
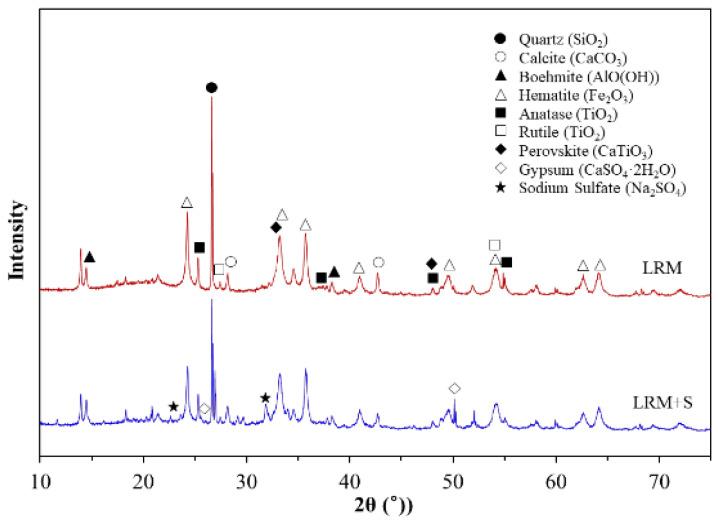
XRD patterns of LRM and LRM + S.

The mineral composition of the LRM + S was analyzed, and the results are shown in Figure 3. The main compounds identified in the LRM were quartz, calcite, boehmite, and hematite [25]. These compounds were also found in the LRM + S. Characteristic peaks were observed in the LRM + S at 2θ = 25.5° and 51.1° owing to the addition of sulfuric acid; they were identified as gypsum and sodium sulfate, respectively.

#### 3.1.3. Physical Properties

Table 5 shows the physical properties of the LRM and LRM + S. The physical properties of the LRM included a water content of 48.6%, pH of 11.5, density of 1.50 g/cm^3^, specific surface area of 2871 m²/kg, average particle diameter of 2.50 µm, and viscosity of 36,670 cP.

The water content of the LRM + S decreased to 44.1% owing to sulfuric acid neutralization, and the viscosity increased significantly to 60,670 cP. The average particle size increased to 3.02 µm, and the specific surface area decreased to 2441 m²/kg.

### 3.2. Flow

**Figure 4:**
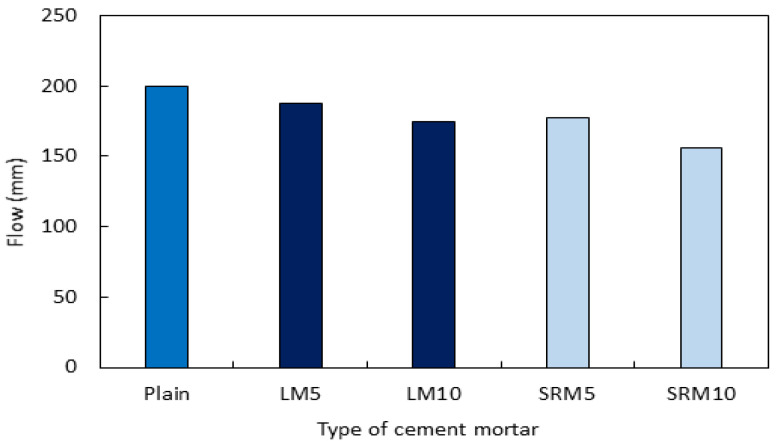
Flows of cement mortars with varying proportions of LRM or LRM + S.

Figure 4 shows the flows of LM and SRM, the cement mortars that contained LRM and LRM + S, respectively. The flows of the LM and SRM cement mortars were lower than that of the plain cement mortar. The flow value decreased as the proportion of red mud in the mix increased. Red mud has a larger specific surface area and lower density than OPC. In addition, the flows of the SRM mortars were lower than those of the LM mortars. This can be attributed to LRM + S having a viscosity that is 1.6 times higher than that of LRM owing to sulfuric acid neutralization. 

### 3.3. Setting Time

**Figure 5:**
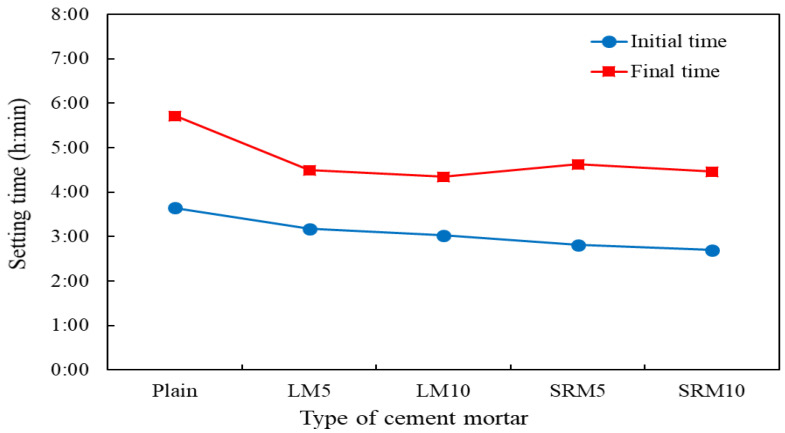
Initial and final setting times of cement mortars with varying proportions of LRM or LRM + S.

Figure 5 shows the setting time measurements’ results on plain, LM, and SRM cement mortars. The setting times of LM and SRM mortars were shortened to that of the plain mortar. In particular, the final setting time was significantly shortened compared to the initial setting time. The initial and final setting times of the LM10 were 37 and 82 min shorter, respectively, than those of plain mortar. The initial and final setting times of the SRM10 were 57 and 75 min shorter, respectively, than those of plain mortar. Liquid red mud is an aluminate-based quick-setting agent that decomposes into alkali (NaOH) and aluminum hydroxide (Al(OH)_3_) in concrete. At this time, the alkali component (NaOH) generated promotes the hydration of calcium silicate (C_2_S, C_3_S) in cement to produce a large amount of calcium hydroxide in the initial stage. 3CaO·Al_2_O_3_·6H_2_O is generated to promote cement setting and hardening, so the setting time of LR and SRM specimens is shown to be shorter than that of plain specimens [26]. Therefore, the setting time can be accelerated when the cement of the cement mortar is replaced with LRM or LRM + S. 

### 3.4. Compressive Strength

**Figure 6:**
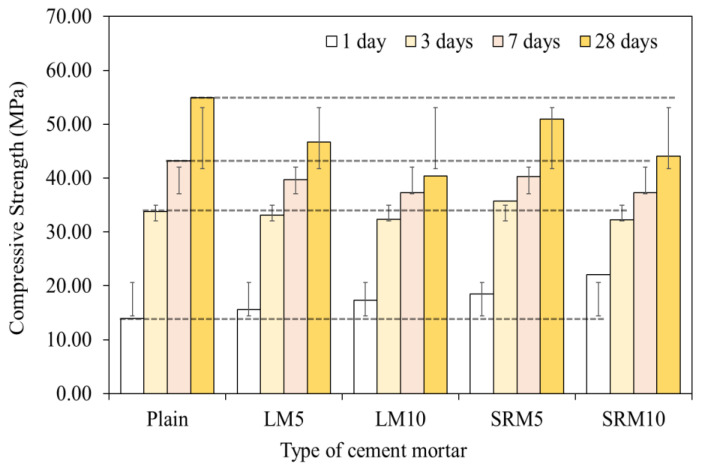
Effects of LRM and LRM + S on compressive strength of the cement mortar.

**Figure 7:**
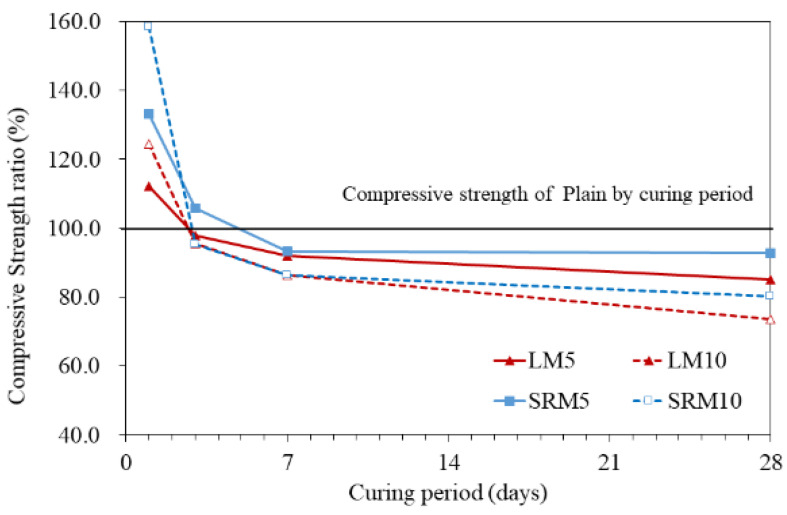
Compressive strength ratios of LRM- and LRM + S-modified cement mortar over the aging period.

Figure 6 and Figure 7 show the compressive strength measurement results and the compressive strength ratios of the LM and SRM mortars compared to that of the plain mortar. The compressive strengths of LM and SRM were initially higher than that of plain and remained so for up to 3 days of aging. After that, their compressive strengths became lower than that of plain mortar. However, the compressive strength of the SRM at 28 days of aging was still higher than that of the LM, confirming that sulfuric acid neutralization can improve compressive strength. The compressive strength decreased further when higher proportions of LRM and LRM + S were substituted.

The compressive strengths after 1 day of aging were found to be 13.92 MPa for plain mortar, 17.32 MPa for LM10, and 22.05 MPa for SRM10. The compressive strengths of the LM10 and SRM10 were approximately 24% and 58% higher than that of plain mortar, respectively. LRM + S was found to contribute significantly to the initial strength. However, the compressive strengths of LM10 and SRM10 after 3 days of aging were similar to that of plain mortar and were lower after 7 days.

While substituting LRM and LRM + S improved the initial compressive strength, this tendency was reversed with longer aging times. After 28 days of aging, the compressive strengths were found to be 54.91 MPa for plain, 40.41 MPa for LM10, and 44.08 MPa for SRM10. The compressive strengths of the LM10 and SRM10 after 28 days of aging were lower than that of plain mortar by approximately 26% and 19%, respectively. However, the compressive strength of the SRM10 was 7% higher than that of the LM10, indicating that the compressive strength was improved by sulfuric acid neutralization.

In previous studies, a maximum red mud content of 10% was proposed considering the reduction in the compressive strength of cement mortar caused by substituting red mud [27,28,29]. The results of this study indicate that red mud content can be increased through sulfuric acid neutralization because LRM + S can significantly improve the initial compressive strength of cement mortar [30].

### 3.5. SEM Observations Instead

**Figure 8:**
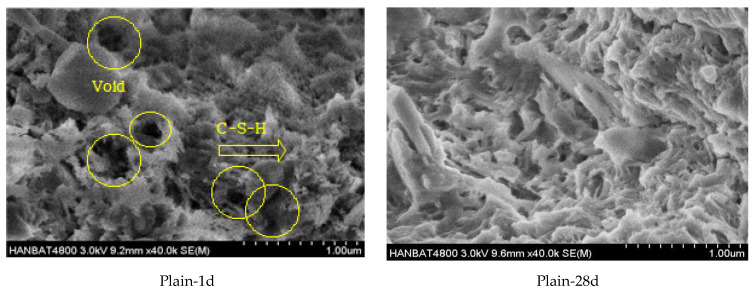

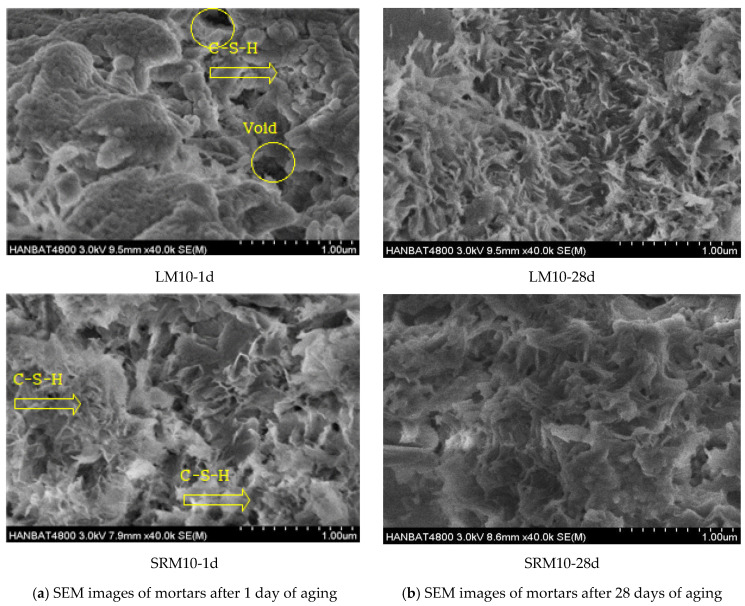
SEM images of Plain, LM10, and SRM10 mortars after (**a**) 1 day and (**b**) 28 days of aging.

Figure 8 shows the SEM images of the microstructures of plain, LM10, and SRM10 after 1 day and 28 days of aging. In Figure 8a, more pores are observed in the microstructure of Plain-1d than in those of LM10-1d and SRM10-1d. This appears to be due to the larger particle size of OPC at 14 µm [31] versus those of LRM and LRM + S at 2.50 and 3.02 µm, respectively. In the LM10 and SRM10, pores were more densely filled with smaller particles. This may be the reason for the higher compressive strength of the LM and SRM compared to that of plain mortar after 1 day of aging. Figure 8b shows that the microstructures became denser as aging increased due to increased hydration products. Plain-28d and SRM10-28d exhibited larger hydration products than LM10-28d. This difference in hydration products could be the reason for the slightly lower compressive strength of LM10 after 28 days.

### 3.6. XRD

**Figure 9:**
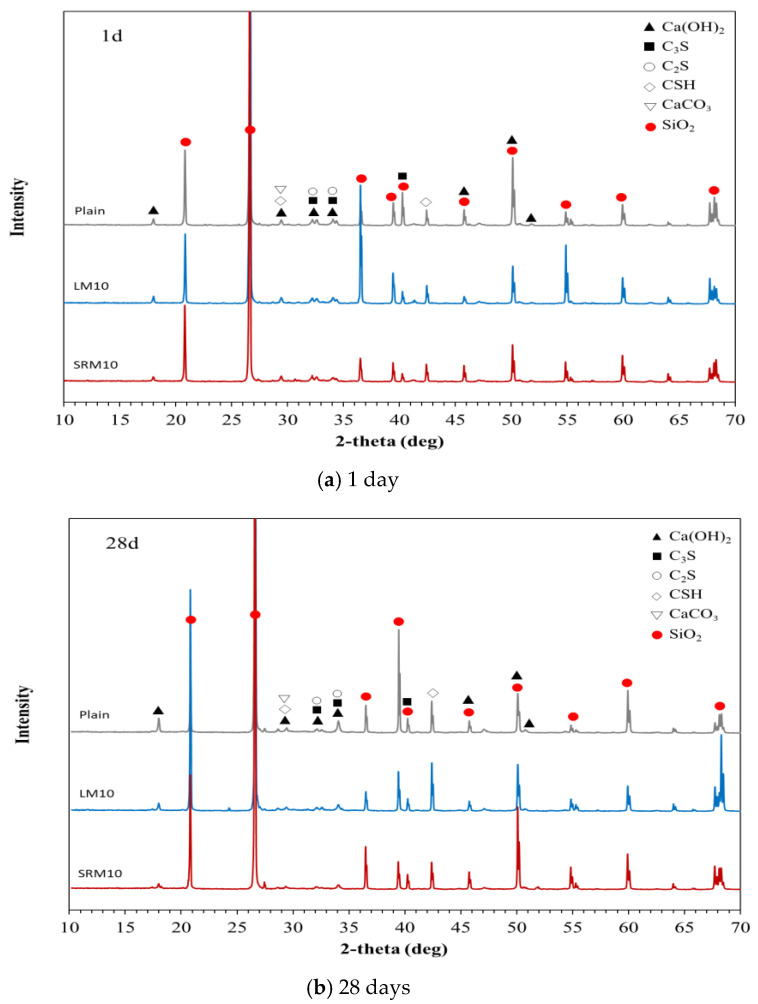
XRD results of the Plain, LM10, and SRM10 mortars after (**a**) 1 day and (**b**) 28 days.

XRD analysis was conducted to identify the hydration products of the plain, LM10, and SRM10 mortars after 1 day and 28 days of aging. The results are shown in Figure 9. Figure 9a shows that the peaks of unhydrated cement particles (C3S and C2S) and hydration products (CSH and CaOH_2_) were observed in Plain-1d [26]. The XRD peaks of the LM-1d and SRM-1d were similar to those of Plain-1d, indicating that the hydration products were identical [32]. Figure 9b shows that the XRD peak patterns of Plain-28d and the SRM10-28d were similar, but the pattern of LM10-28d exhibited a new peak at 2θ = 24°, which is judged to be xonotlite [33,34]. This coincides with the SEM images in Figure 8b, which show that LM10-28d had a different microstructure to those of Plain-28d and SRM10-28d, indicating hydration products with a fine fibrous structure.

## 4. Conclusions

This study investigated the feasibility of using LRM and LRM + S as cement substitutes in cement mortar to increase the recycling rate of LRM. LRM was obtained by dispersing red mud generated from aluminum production in water, without drying or crushing, resulting in an LRM with a water content of approximately 35 wt.%. LRM + S was obtained by neutralizing the LRM with sulfuric acid. LRM and LRM + S were then added into cement mortar as cement substitutes, and the hydration and mechanical characteristics of the mortars were investigated. The following conclusions were drawn.

1. When LRM was neutralized with sulfuric acid and stabilized to a pH of 7 to 8, gypsum and sodium sulfate were generated, and the physical properties of the LRM, including particle size, specific surface area, and viscosity, were changed. In particular, the viscosity increased by 1.6 times.
2. Substituting LRM and LRM + S for cement in cement mortar decreased the flow. The flow value decreased as the amount of red mud increased. The measurement results showed that the setting times of LM and SRM, which contained LRM and LRM + S, were shorter than those of plain mortar. In particular, the final setting times were significantly shortened.
3. The compressive strengths of LM and SRM were initially higher than that of plain mortar but decreased with aging. The compressive strengths of the LM10 and SRM10 were approximately 24% and 58% higher than that of plain mortar, respectively, after 1 day of aging. After approximately 3 days of aging, the compressive strengths of the LM10 and SRM10 were comparable to that of plain mortar. After 28 days of aging, it was lower than plain mortar by approximately 26 and 19%, respectively. The compressive strength decreased as the amounts of LRM and LRM + S in the mortar increased. The compressive strength of SRM at 28 days was higher than that of LM, confirming the compressive strength improvement effect of sulfuric acid neutralization.
4. More pores were observed in the microstructure of the plain mortar compared to those of the LM10 and SRM10 mortars at 1 day of aging. This can explain the higher initial compressive strengths of LM and SRM compared to that of plain mortar. At 28 days of aging, the plain and SRM10 mortars exhibited similar microstructures, unlike the LM10 mortar, which exhibited a fine fibrous structure. This difference in hydration products can explain the slightly lower compressive strength of LM10 after 28 days of aging. The XRD analysis showed that the hydration products of the plain, LM10, and SRM10 mortars were identical at 1 day of aging. XRD indicated the formation of xonotlite in the LM10 after 28 days of aging.

The results of this study show that it may be possible to increase the recycling rate of red mud in cement concrete by neutralization with sulfuric acid. Studies on the hydration mechanisms and long-term strength and durability will be required.

## Figures and Tables

**Table 1:** Properties of red mud sludge.

Type	Chemical Composition (wt.%)								Moisture Content Ratio (wt.%)
	SiO_2_	Al_2_O_3_	Fe_2_O_3_	CaO	MgO	SO_3_	Na_2_O	K_2_O	
Red mud sludge	38.8	16.1	22.8	3.4	0.21	0.29	10.0	0.4	36

**Table 2:** Physical properties and chemical composition of OPC.

Type	Blaine (cm^2^/g)	Setting Time		Density (g/cm^3^)	Chemical Composition (wt.%)						
		Initial (min)	Final (h)		SiO_2_	Al_2_O_3_	Fe_2_O_3_	CaO	MgO	SO_3_	Lg. Loss
OPC *	3300	200	5.5	3.15	21.7	5.7	3.2	63.1	2.8	2.2	1.3

* OPC: Ordinary Portland Cement.

**Table 3:** Properties of Admixtures.

Type	Color	Specific Gravity	pH	Residue Content (%)	Viscosity (cP)	NaCl (wt.%)	Moisture (wt.%)
Polycarboxylic acid series	Light brown	1.136	6.72	40.7	180	-	-
Methyl cellulose	White	-	-	-	32,900	1.36	1.40

**Table 4:** Mix design.

Mix ID	Mix Design (g)				
	Cement	Sand	Water	LRM	LRM + S
Plain	100	300	50	-	-
LM5 *	95		45.27	9.72	-
LM10	90		40.54	19.46	-
SRM5 **	95		46.06	-	8.94
SRM10	90		42.11	-	17.89

* Liquefied red mud +Mortar ** Sulphuric neutralization liquid red mud + Mortar.

**Table 5:** Physical properties of LRM.

Type of Red Mud	Moisture Content (%)	pH	Density (g/cm^3^)	Viscosity (cP)	Average Particle Size (µm)	Specific Surface Area (m²/kg)
LRM	48.6	11.5	1.50	36,670	2.50	2871
LRM + S	44.1	7.6	1.54	60,670	3.02	2441

## Data Availability

The data presented in this study are available on request from the corresponding author.

## References

[1] Jiang T., Cui K., Chang J. (2023). Development of low-carbon cement: Carbonation of compounded C2S by β-C2S and γ-C2S. Cem. Concr. Compos..

[2] Wang L., Chen L., Provis J.L., Tsang D.C.W., Poon C.S. (2019). Accelerated carbonation of reactive MgO and Portland cement blends under flowing CO_2_ Gas. Cem. Concr. Compos..

[3] Cui K., Liang K., Jiang T., Zhang J., Lau D., Chang J. (2023). Understanding the role of carbon nanotubes in low-carbon concrete: From experiment to molecular dynamics. Cem. Concr. Compos..

[4] Cui K., Lu D., Jiang T., Zhang J., Jiang Z., Zhang G., Lau D. (2023). Understanding the role of carbon nanotubes in low carbon sulfoaluminate cement-based composite. J. Clean. Prod..

[5] Lima M.S.S., Thives L.P. (2020). Evaluation of red mud as filler in Brazilian dense graded asphalt mixtures. Constr. Build. Mater..

[6] Menzie W.D., Barry J.J., Bleiwas D.I., Bray E.L., Goonan T.G., Matos G. (2010). The Global Flow of Aluminum from 2006 through 2025.

[7] Liu X., Zhang N., Yao Y., Sun H., Feng H. (2013). Micro-structural characterization of the hydration products of bauxite-calcination-method red mud-coal gangue based cementitious materials. J. Hazard. Mater..

[8] Hildebrando E.A., Souza J.A.d.S., Angélica R.S., Neves R.F. (2013). Application of bauxite waste from Amazon region in the heavy clay industry. Mater. Res..

[9] Nath H., Sahoo A. (2014). A study on the characterization of red mud. Int. J. Appl. Bio-Eng..

[10] Khairul M.A., Zanganeh J., Moghtaderi B. (2019). The composition, recycling and utilisation of Bayer red mud. Resour. Conserv. Recy..

[11] Cui K., Chang J. (2022). Hydration, reinforcing mechanism, and macro performance of multi-layer graphene-modified cement composites. J. Build. Eng..

[12] Wang S., Ang H.M., Tadé M.O. (2008). Novel applications of red mud as coagulant, adsorbent and catalyst for environmentally benign processes. Chemosphere.

[13] Zhou J., Ma S., Chen Y., Ning S., Wei Y., Fujita T. (2021). Recovery of scandium from red mud by leaching with titanium white waste acid and solvent extraction with P204. Hydrometallurgy.

[14] Bonomi C., Alexandri A., Vind J., Panagiotopoulou A., Tsakiridis P., Panias D. (2018). Scandium and titanium recovery from bauxite residue by direct leaching with a Brønsted acidic ionic liquid. Metals.

[15] Li Z., Afshinnia K., Rangaraju P.R. (2016). Effect of alkali content of cement on properties of high performance cementitious mortar. Constr. Build. Mater..

[16] Muraleedharan M., Nadir Y. (2021). Factors affecting the mechanical properties and microstructure of geopolymers from red mud and granite waste powder: A review. Ceram. Int..

[17] Kang S., Kang H., Lee B. (2021). Hydration properties of cement with liquefied red mud neutralized by nitric acid. Materials.

[18] Kang S., Kang H., Lee B. (2020). Effects of adding neutralized red mud on the hydration properties of cement paste. Materials.

[19] Kang S.P., Kwon S.J. (2017). Effects of red mud and alkali-activated slag cement on efflorescence in cement mortar. Constr. Build. Mater..

[20] Liu X., Zhang N., Sun H., Zhang J., Li L. (2011). Structural investigation relating to the cementitious activity of bauxite residue—Red Mud. Cem. Concr. Res..

[21] Choe G., Kang S., Kang H. (2020). Mechanical properties of concrete containing liquefied red mud subjected to uniaxial compression loads. Materials.

[22] (2020). Standard Test Method for the Flow of Hydraulic Cement Mortar.

[23] (2021). Standard Test Method for Time of Setting of Hydraulic Cement by Vicat Needle.

[24] (2018). Test Method (Standard Test Method for Compressive Strength of Hydraulic Cement Mortars.

[25] Choe G., Kang S., Kang H. (2019). Characterization of slag cement mortar containing nonthermally treated dried Red Mud. Appl. Sci..

[26] Ribeiro D.V., Labrincha J.A., Morelli M.R. (2011). Potential use of natural red mud as pozzolan for Portland cement. Mater. Res..

[27] Niu M., Li G., Zhang J., Cao L. (2020). Preparation of alkali-free liquid accelerator based on aluminum sulfate and its accelerating mechanism on the hydration of cement pastes. Constr. Build. Mater..

[28] Kunther W., Lothenbach B., Scrivener K. (2013). Influence of bicarbonate ions on the deterioration of mortar bars in sulfate solutions. Cem. Concr. Res..

[29] Ortega J., Cabeza M., Tenza-Abril A.J., Real-Herraiz T., Climent M.Á., Sánchez I. (2019). Effects of red mud addition in the microstructure, durability and mechanical performance of cement mortars. Appl. Sci..

[30] Senff L., Modolo R.C.E., Silva A.S., Ferreira V.M., Hotza D., Labrincha J.A. (2014). Influence of red mud addition on rheological behavior and hardened properties of mortars. Constr. Build. Mater..

[31] Jawad Z.F., Ghayyib R.J., Salman A.J. (2020). Microstructural Analysis for Cement Mortar with Different Nano Materials. Mater. Sci. Forum.

[32] He Y., Zhang X., Liu S., Hooton R.D., Ji T., Kong Y. (2020). Impacts of sulphates on rheological property and hydration performance of cement paste in the function of polycarboxylate superplasticizer. Constr. Build. Mater..

[33] Wu Z., Li L., Gao F., Zhang G., Cai J., Cheng X. (2022). Resource utilization of red mud from the Solid Waste of aluminum Industry Used in Geothermal Wells. Materials.

[34] Van N.D., Imasawa K., Hama Y. (2022). Influence of hydrothermal synthesis conditions and carbonation on physical properties of xonotlite-based lightweight material. Constr. Build. Mater..

